# Allergen immunotherapy and dupilumab in atopic dermatitis: Clinical efficacy and disparities in immunological indicators^☆^

**DOI:** 10.1016/j.waojou.2025.101043

**Published:** 2025-03-12

**Authors:** Jin Liu, Lin Yang, Qingxiu Xu, Qing Jiang, Nan Huang, Wenjing Li, Yaqi Yang, Dongxia Ma, Le Li, Yangxue Fu, Hao Chen, Rongfei Zhu

**Affiliations:** a Department of Allergy, Tongji Hospital, Tongji Medical College, Huazhong University of Science and Technology, China; b Institute of Allergy and Clinical Immunology, Tongji Hospital, Tongji Medical College, Huazhong University of Science and Technology, 1095, Jiefang Avenue, Wuhan, 430030, China

**Keywords:** Atopic dermatitis, Allergen-specific immunotherapy, Dupilumab, Lymphocyte, Immunoglobulin

## Abstract

**Objective:**

Allergen immunotherapy (AIT) and dupilumab have been confirmed to improve symptoms of atopic dermatitis (AD); however, the precise immune mechanisms underlying their efficacy and whether they can elicit synergistic immune effects remain not fully elucidated. We aimed to investigate the clinical efficacy and immunological changes in AD patients undergoing AIT, dupilumab, and a combination of AIT and dupilumab treatment.

**Methods:**

Clinical data, serum samples, and peripheral blood mononuclear cells (PBMC) were collected from house dust mite (HDM)-sensitized AD patients receiving AIT and/or dupilumab at baseline and 6 months. Changes in clinical efficacy, HDM-specific IgE and IgG_4_, serum cytokines, and lymphocyte subgroups were compared among the treatment groups.

**Results:**

A total of 77 AD patients were included, with 39 in the AIT group, 19 in the dupilumab group, and 19 in the AIT combined dupilumab group. The SCORAD scores significantly improved in all groups after 6 months. Levels of HDM-specific IgE and total IgE remained stable in the AIT group but decreased in the dupilumab and combination groups. Levels of IgG_4_ against major mite components *Der p1* and *Der p23* increased in the AIT group and combined treatment group. Serum cytokine levels showed no significant changes, except for a decrease in CCL17 in the dupilumab group. Th1/Th2 and Th17/Th2 ratios increased after dupilumab treatment. There were notable differences in T cell subpopulations when PBMCs were stimulated with HDM extracts after 6-month treatment, tSNE analysis showed the proportion of IL-4^+^IL-13^+^CRTH2^+^T cells increased in the dupilumab group but had no changes in the AIT and combination group.

**Conclusions:**

AIT, dupilumab, and their combination improved clinical symptoms and quality of life in AD patients. AIT promoted allergen-specific IgG_4_ production, while dupilumab modulated T cell responses and reduced allergen-specific IgE synthesis. The combination of AIT and dupilumab exhibited the immunological parameter changes characteristic of both treatments but did not result in a significantly greater improvement in AD symptoms.

## Introduction

Atopic dermatitis (AD) is a common inflammatory skin condition characterized by itching, localized eczema, and dry skin.^1^ The global incidence and prevalence of AD have been increasing in recent decades, affecting around 15%–20% of children and 3%–10% of adults worldwide.^1^^,^^2^ Notably, China has exhibited the highest rates of AD based on the 2019 global burden of disease (GBD) data,^3^ with a 25.65% rise in AD cases from 1990 to 2019^4^ and a lifetime prevalence of 10%–30% among children.^5^ The chronic and recurrent nature of AD leads to significant burdens on patients and their families,^2^ with a high recurrence rate of 75.9% within 7 years.^6^

Various factors contribute to the development of AD, including genetic predisposition, impaired skin barrier function, microbial imbalances, immune dysregulation, and environmental triggers.^1^ Activation of the Th2 pathway is a key feature of AD, with IL-4 and IL-13 playing crucial roles in promoting inflammation.^7^^,^^8^ These cytokines can directly impact epithelial cells, resulting in altered protein expression and increased keratinocyte proliferation. Additionally, they can stimulate B-cell immunoglobulin class switching and IgE production. Approximately 80% of AD cases present as extrinsic AD, characterized by elevated serum IgE levels and impaired skin barrier function. Exposure to airborne, food, and microbial allergens can exacerbate symptoms in these individuals.^9^, ^10^, ^11^ House dust mites (HDM) are the primary allergens for AD. In contrast, about 20% of AD cases manifest as intrinsic AD with normal serum IgE levels, where disease exacerbation is not linked to allergen exposure.^9^, ^10^, ^11^

Topical treatments such as topical corticosteroids (TCS) and topical calcineurin inhibitors (TCI) are the first-line options for AD. However, approximately 30% of patients do not respond adequately to topical treatments and are classified as having moderate-to-severe AD,^12^ necessitating systemic treatment. Dupilumab, an anti-IL-4 receptor alpha subunit antibody, has been approved for treating moderate to severe AD,^13^ showing symptom improvement in various studies.^14^^,^^15^ Furthermore, allergen immunotherapy (AIT) is the only etiological treatment capable of inducing long-term immune tolerance in allergic diseases. AIT can rebalance Th1/Th2 responses and promote the production of protective antibodies.^16^^,^^17^ AIT has demonstrated effectiveness in improving symptoms in HDM-sensitized or polysensitized AD patients in numerous studies. AIT is recommended as an adjunct to topical medications for patients with refractory moderate to severe AD with allergens.^18^ However, the comprehensive understanding of AIT and dupilumab mechanisms in AD remains limited, requiring further research on their combined immunological effects to enhance treatment outcomes and minimize adverse reactions in AD patients. Therefore, we analyzed the clinical efficacy, specific immunoglobulin E (sIgE) and specific immunoglobulin G_4_ (sIgG_4_) to HDM components, serum cytokines, and peripheral blood lymphocyte subsets of AD patients receiving AIT, dupilumab, and AIT combined with dupilumab. Biomarkers associated with different treatment responses were also explored.

## Methods

### Study population

The study population was selected from AD patients who received AIT and/or dupilumab at —Tongji Hospital from August 2022 to May 2023. The inclusion criteria included: (1) age≥3 years old; (2) meet the diagnostic criteria of Hanifin and Rajka, and diagnosed as AD; (3) serum dust mite specific IgE≥0.35kU_A_/L; and (4) no contraindications for AIT and/or dupilumab. The exclusion criteria included: (1) combined with other skin condition except AD; (2) had chronic systemic diseases, such as diabetes, coronary heart disease, cerebrovascular disease, autoimmune diseases, malignant tumors; (3) pregnancy or lactation; and (4) use of other systemic medications that could affect the immune system within 3 months before or during treatment. The patients were categorized into 3 groups based on their treatment: the AIT group, the dupilumab group, and the AIT combined — dupilumab group. AIT and dupilumab represent distinct systemic treatment approaches for AD. AIT offers a disease-modifying effect with demonstrated long-term efficacy, whereas dupilumab is effective for rapid symptom control. Consequently, we introduced both treatments to patients sensitized to HDM and empowered them to make the final decision regarding their treatment regimen. This included the option to choose AIT, dupilumab, or a combination of both based on their individual needs.

Clinical symptom assessment and serological detection were performed at baseline and 6 months after treatment. This study was approved by the Ethics Committee of Tongji Hospital (Ethics approval number: xxxxxxTJ IRB20231112) and strictly implemented in accordance with the relevant principles of the Declaration of Helsinki. All the patients and/or their guardians had signed the informed consent.

### Allergen immunotherapy

Patients in the AIT group received subcutaneous immunotherapy (SCIT) for 6 months. The dose building-up phase lasts for 15 weeks, and patients were required to inject AlutardSQ (ALK-Abello A/S, Denmark) once a week in the outpatient clinic. The concentration of injection was from 100SQ-U/mL, 1000SQ-U/mL to 10000SQ-U/mL, successively, with 0.2, 0.4, and 0.8 mL each dose; 100000SQ-U/mL was injected with the doses of 0.1, 0.2, 0.4, 0.6, 0.8, and 1.0 mL, successively. In the dose maintenance phase, 1.0 mL was injected every 4–6 weeks.

### Dupilumab

Patients in the dupilumab group received subcutaneous injection of dupilumab (Sanofi Biotechnology, France) for 6 months. The injection dose is selected according to the manufacturer's instructions: patients with AD receive an initial dose of 600 mg (>18 years old) or 300 mg (<18 years old), and thereafter every 2 weeks (>18 years old) or every 4 weeks (<18 years old) with 300 mg each injection.

### Allergen immunotherapy combined with dupilumab

Patients in the AIT combined dupilumab group received both AIT and dupilumab simultaneously for 6 months. AIT and dupilumab were administered in the same manner as described.

Patients are allowed to use topical drugs such as moisturizer during the treatment. Topical glucocorticoids, calcineurin inhibitors, and oral antihistamines can be used as rescue medication during AIT and/or dupilumab treatment.

### Clinical evaluation

The demographic data and clinical information of AD patients was collected at baseline. Scoring atopic dermatitis index (SCORAD), patient-oriented eczema measure (POEM), itch numerical rating scale (Itch-NRS), atopic dermatitis control test (ADCT), and the dermatology life quality index (DLQI) were evaluated at baseline and 6 months after treatment.^19^ Besides, the total combined symptom and medication score (CSMS, which is the sum of rhinitis symptom and medication score; range from 0 to 6)^20^ was used for evaluation of nasal symptoms in patients with comorbid AR.

### tIgE, specific IgE, and IgG4 to HDM components

Serum total IgE (tIgE) levels (detection range: 2-5000KU/L) and *Der p*-sIgE and *Der f*-sIgE levels (detection range: 0-100kU_A_/L) were measured by ImmunoCap. The sIgE and sIgG_4_ to *Der p* components *Der p1*, *Der p2*, *Der p5*, *Der p7*, *Der p10*, *Der p21*, *Der p23* and *Der f* components *Der f1* and *Der f2* were detected by protein chip technology (Hangzhou Zheda Dixun Biological Gene Engineering Co., Ltd.). The detection principle and procedure of sIgG_4_ are the same as sIgE.

### Cytokines

Serum levels of IL-4, IL-13, IL-17A, CCL17, IL-10, and IFN-γ in AD patients were measured using QuantiCyto®ELISA series products (Xinbosheng Biotechnology Co., Ltd.) according to the principle of enzyme-linked immunosorbnent assay (ELISA). The OD450 value was measured on the enzyme-linked immunosorbent apparatus at 450 nm.

### Peripheral blood mononuclear cells (PBMC)

**Fig. S1:**
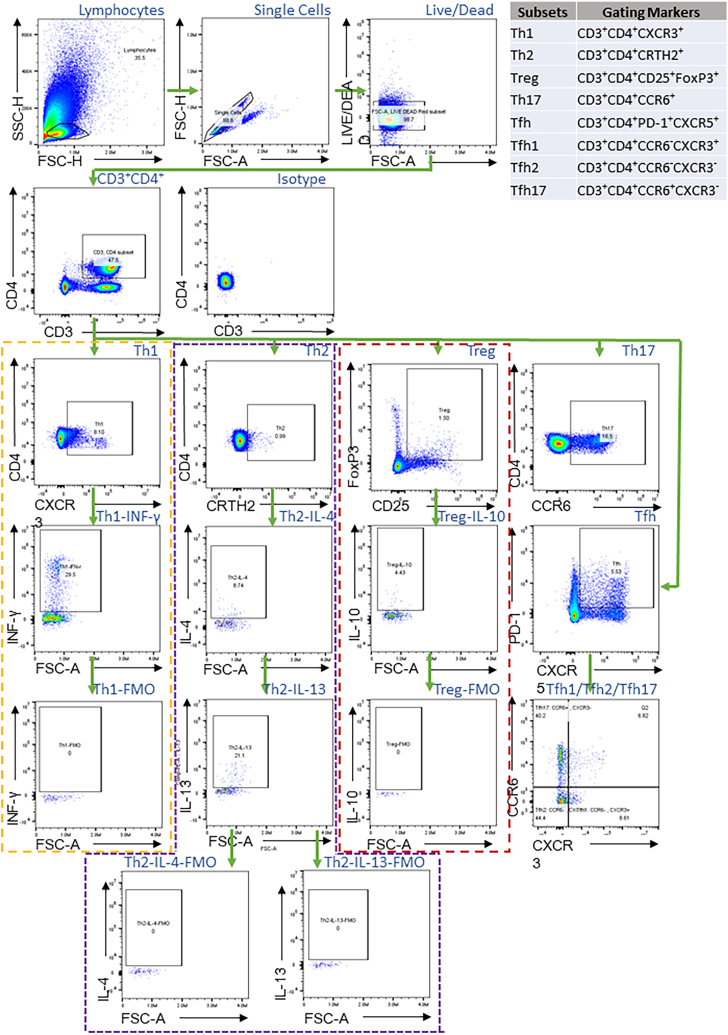
The gating strategy of CD3^+^CD4^+^live T cells.

The PBMCs, which were stored in liquid nitrogen, were thawed in a 37 °C water bath, and then washed and prepared in 100 μl fluorescence-activated cell sorting (FACS) staining buffer at a concentration of 1 × 10ˆ6/mL. Surface staining was performed with anti-human CD3, CD4, CD25, CD127, CD279(PD-1), CD183 (CXCR3), CD185 (CXCR5), CD294 (CRTH2), and CCR6. PBMCs were incubated with 20 μg/mL of HDM extracts for 7 days, adding 25 ng/mL of phorbol 12-myristate 13-acetate (PMA) for the last 4 h. At the end of incubation, the harvested cells were prepared in 100 μl FACS staining buffer at the concentration of 1 × 10ˆ6/mL. Permeabilization buffer was used to increase the permeability of the cell membrane. Intracellular staining was performed with anti-human IL-4, IL-10, IL-13, IFN-γ, and FoxP3. Stained cells were analyzed using FACS Celesta flow cytometry and data were analyzed using Flowjo 10.8.1 software (Treestar, Ashland, OR). Flow cytometry was used to analyze the proportion of CD4^+^T cell subsets (Fig. S1). The CD3^+^CD4^+^live T cells were divided into 5 subgroups: Th1 cells (CXCR3^+^CCR6^-^), Th2 cells (CRTH2^+^), Th17 cells (CCR6^+^), Treg cells (CD25^+^CD127^-^), and Tfh cells (CXCR5^+^PD-1^+^). Tfh cells were further divided into 3 subgroups: Tfh1 cells (CXCR3^+^CCR6^-^), Tfh2 cells (CXCR3^−^CCR6^-^), and Tfh17 cells (CXCR3^−^CCR6^+^). We set unstained case as blank control, mouse anti-human IgG2a staining as isotype and FMO of membrane staining for intracellular cytokine detection.

### tSNE, FlowSOM algorithm, and cluster explorer

The tSNE and FlowSOM algorithm is consistent with our previous research approach.^21^ Viable cells were down sampled to 3600 cells for CD3^+^CD4^+^live cells per group using the Downsample version 3.3 plugin for FlowJo and were concatenated to 1 sample per group. FlowSOM was performed for each study group separately. CD3^+^CD4^+^live cells were sorted into 12 metaclusters, which provided sufficient metaclusters to capture the expected number of unique cell types while potentially uncovering other biologically interesting populations. The Cluster Explorer plugin was used after tSNE and FlowSOM to create an interactive cluster Profile graph, heatmap, and displays the cluster populations on a tSNE/UMAP plot.

### Statistical analysis

SPSS 26.0 software and GraphPad Prism 9.0 software (GraphPad software, San Diego, CA, USA) were used for statistical analysis. Shapiro-Wilk test was used for normality test. For continuous variables, normally distributed data was expressed as mean ± SD, while non-normally distributed data were expressed as interquartile ranges (IQRs, P_25_, P_75_). The categorical variables were expressed as percentages. Differences between groups were compared, χ2 or Fisher's exact test was used for associations between categorical variables, and Mann-Whitney *U* test or Kruskal-Wallis H rank sum test was used for continuous variables that did not obey normal distribution. *P* < 0.05 was considered statistically significant.

## Results

### Dermagraphic characteristics

**Fig. 1:**
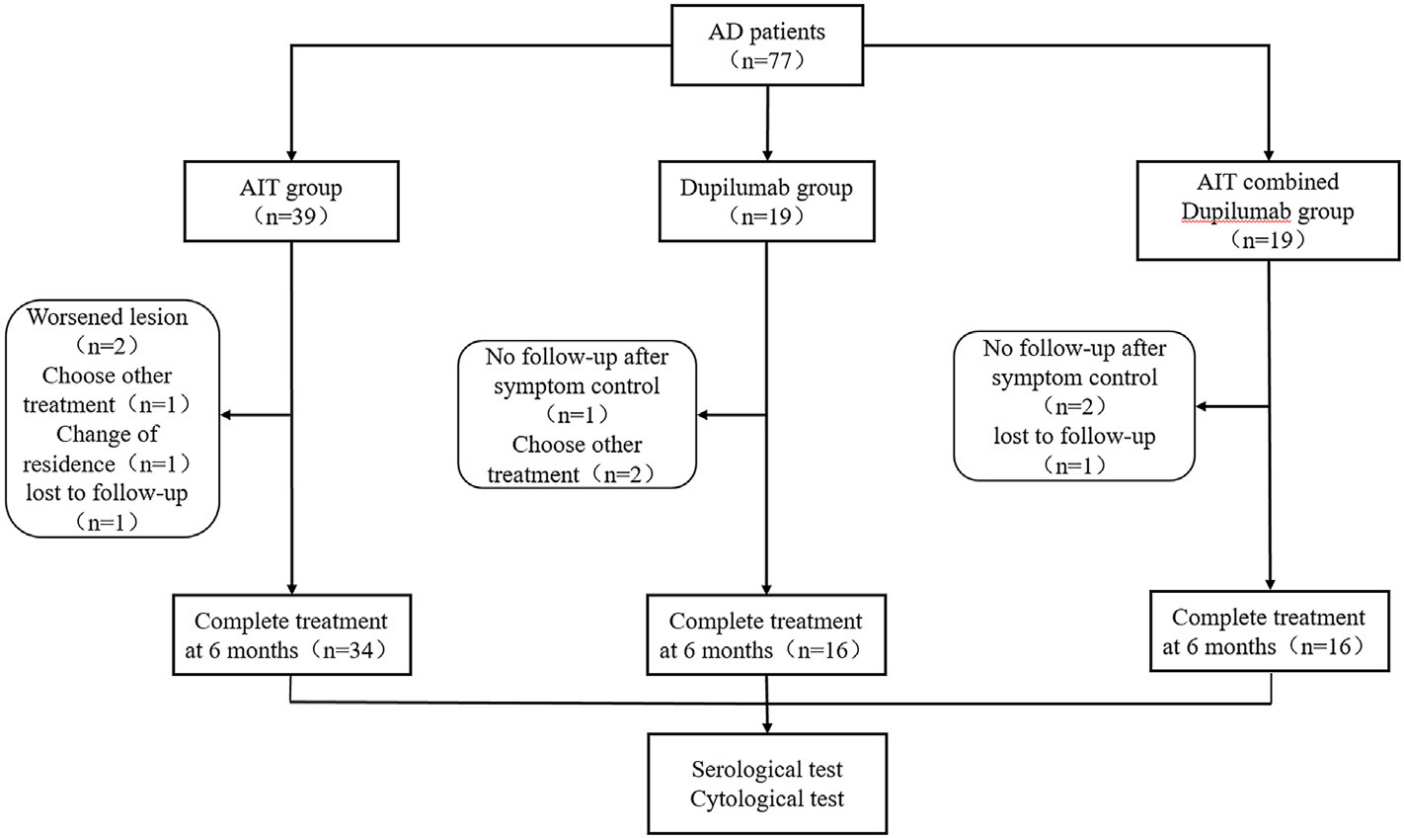
**Follow-up flow chart of AD patients.** AD, atopic dermatitis; AIT, allergen immunotherapy

A total of 77 patients with HDM sensitized AD were enrolled in this study, including 39 in the AIT group, 19 in the dupilumab group, and 19 in the AIT combined dupilumab group. A total of 11 patients dropped out of the study (dropout rate: 14.29%) (Fig. 1). The dermographic characteristics of AD patients in the 3 groups were shown in Table 1. According to the baseline SCORAD score, the severity of AD patients was classified into mild (SCORAD<25), moderate (25≤SCORAD<50), and severe (SCORAD≥50).^22^^,^^23^

**Table 1:** The baseline demographics and disease characteristics in 3 treatment groups.

	AIT group (n = 39)	Dupilumab group (n = 19)	AIT combined Dupilumab group (n = 19)	*P* value
Age (year)	9.78 ± 5.70	9.68 ± 8.73	15.65 ± 13.12	0.1216
Sex (female), *n* (%)	13 (33.22)	9 (47.37)	4 (21.10)	0.1186
AD duration (year)	3.70 ± 2.17	5.21 ± 5.14	6.13 ± 5.02	0.1393
Atopic comorbidities, *n* (%)				
None	4 (10.26)	8 (40.0)	9 (45.0)	/
Food allergies	2 (5.12)	4 (21.05)	3 (15.0)	/
Asthma	12 (30.77)	3 (15.79)	3 (15.0)	/
Allergic rhinitis	35 (89.74)	10 (52.63)	9 (45.0)	/
Family history, *n* (%)	21 (53.85)	7 (36.84)	12 (63.16)	0.7092
Allergen, *n* (%)				
Mono-sensitization	7 (17.95)	8 (42.11)	5 (26.32)	0.1438
Multi-sensitization	32 (82.05)	11 (57.89)	14 (73.68)	0.1438
SCORAD∗∗∗∗^a^^,^^b^	29.66 ± 15.32	55.38 ± 19.23	46.46 ± 18.09	0.0001
POEM∗∗∗^b^	11.87 ± 7.62	20.32 ± 6.83	17.05 ± 6.07	0.0004
Itch-NRS∗∗∗^b^	5.31 ± 2.38	7.84 ± 1.34	6.32 ± 2.63	0.0005
ADCT∗∗∗^b^	10.32 ± 6.14	17.16 ± 5.46	13.26 ± 5.96	0.0008
DLQI∗^b^	6.69 ± 4.63	12.11 ± 8.33	9.74 ± 4.61	0.0096
Severity of AD, *n* (%)				
Mild∗∗^a^^,^^b^	17 (43.59)	1 (5.26)	1 (5.26)	0.0005
Moderate	16 (41.03)	7 (36.84)	8 (42.11)	0.9377
Severe∗∗^a^^,^^b^	6 (15.38)	11 (57.89)	10 (52.63)	0.0011
tIgE, KU/L	835.10 ± 714.20	1445.00 ± 2005.00	1386.00 ± 1553.00	0.3611
*Der p*-sIgE, kU_A_/L	53.36 ± 35.30	40.95 ± 38.93	58.79 ± 38.03	0.4644
*Der f*-sIgE, kU_A_/L	71.10 ± 36.01	47.44 ± 42.61	70.11 ± 36.31	0.1250

Data are presented as numbers or mean ± SD. Fisher's exact test was used for comparison of categorical variables, and Kruskal-Wallis test was used for comparison of continuous variables. ∗P < 0.05, ∗∗P < 0.01, ∗∗∗P < 0.001, ∗∗∗∗P < 0.0001 when compared between AIT group and Dupilumab group.

—

a P < 0.01 when compared between AIT group and AIT combined Dupilumab group.

b P > 0.05 when compared between Dupilumab group and AIT combined Dupilumab group. AIT, allergen immunotherapy; SCORAD, scoring atopic dermatitis index; POEM, patient-oriented eczema measure; Itch-NRS, itch numerical rating scale; ADCT, atopic dermatitis control test; DLQI, dermatology life quality index; tIgE, total IgE; sIgE, specific IgE; *Der p*, *Dermatophagoides pteronyssinus*; *Der f*, *Dermatophagoides farina*

### Clinical efficacy

#### Effectiveness

**Fig. 2:**
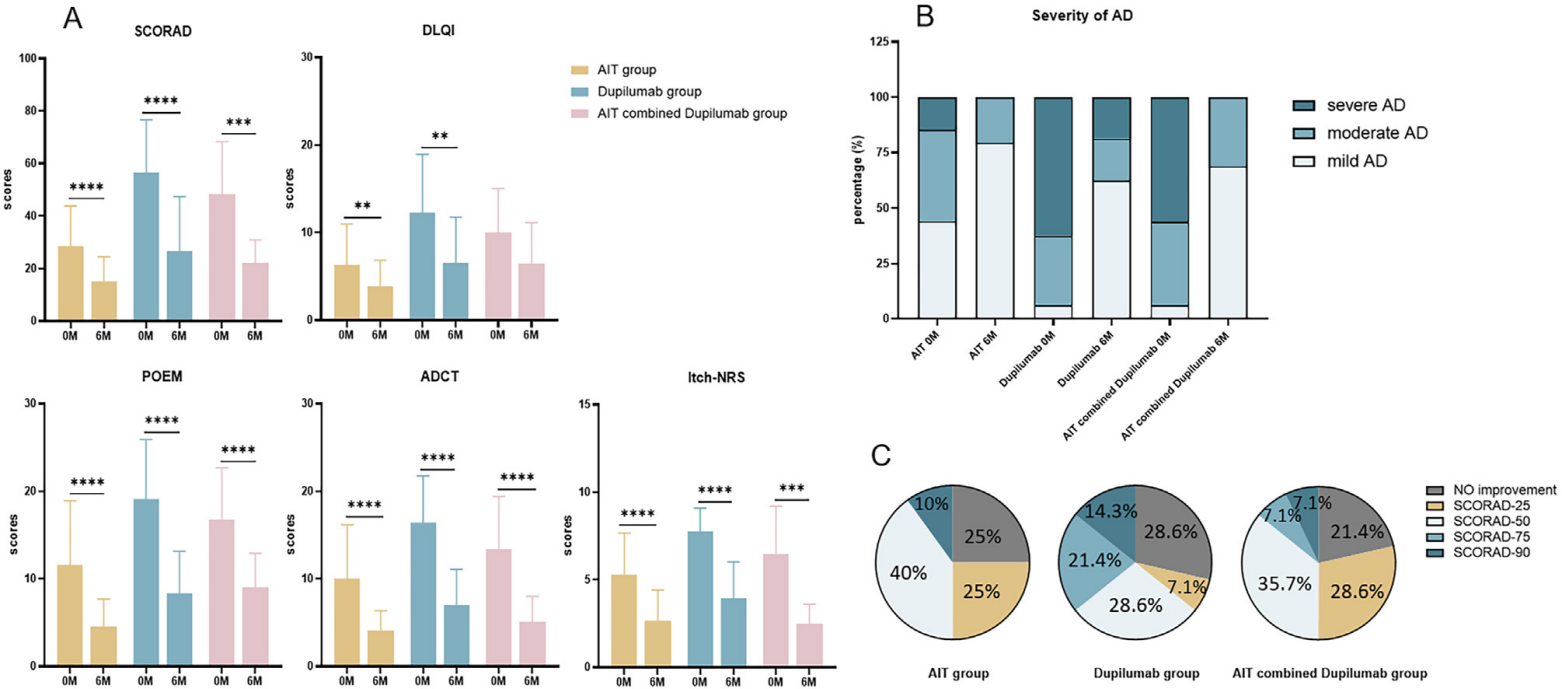
**Clinical symptom improvement of AD patients in** 3 **treatment groups.** (A) Clinical symptom scores at baseline and 6 months in 3 treatment groups. (B) Proportion of patients with mild, moderate, and severe AD at baseline and 6 months in 3 treatment groups. (C) The proportion of patients achieved 5 degrees of improvement as SCORAD-90 (ΔSCORAD≥90%), SCORAD-75 (90%>ΔSCORAD≥75%), SCORAD-50 (75%>ΔSCORAD≥50%), SCORAD-25 (50%>ΔSCORAD≥25%) and no improvement (ΔSCORAD<25%) in 3 treatment groups. Columns with error bars represented the mean with SD. Wilcoxon test ∗∗P < 0.01; ∗∗∗P < 0.001; ∗∗∗∗P < 0.0001. AD, atopic dermatitis; AIT, allergen immunotherapy; SCORAD, scoring atopic dermatitis index; POEM, patient-oriented eczema measure; Itch-NRS, itch numerical rating scale; ADCT, atopic dermatitis control test; DLQI, dermatology life quality index

The SCORAD, POEM, Itch-NRS, ADCT, and DLQI scores of AD patients in the AIT group and dupilumab group significantly decreased after treatment compared with baseline (P < 0.01) (Fig. 2A). The SCORAD, POEM, Itch-NRS, and ADCT scores of AD patients in the AIT combined dupilumab group significantly decreased after treatment compared with baseline (P < 0.001) (Fig. 2A). The proportion of patients with moderate and severe AD decreased in all groups after treatment (Fig. 2B). There were no significant differences in the improvement rates of SCORAD, POEM, Itch-NRS, ADCT, and DLQI among the 3 groups (Table 2). There were 50.0%, 64.3%, 49.9% of patients achieving at least 50% improvement of SCORAD in the AIT group, dupilumab group, and AIT combined dupilumab group, respectively (Fig. 2C). After 6 months of treatment, the CSMS significantly decreased in the AIT group (3.28–1.79, P < 0.0001), Dupilumab group (3.20–1.70, P = 0.0078) and AIT combined Dupilumab group (3.56–1.67, P = 0.0039). There was no significant difference in CSMS among the 3 groups at baseline and posttreatment (P > 0.05).

**Table 2:** Clinical symptom scores at baseline and 6 months in 3 treatment groups.

	AIT group (n = 34)	Dupilumab group (n = 16)	AIT combined Dupilumab group (n = 16)	P value
SCORAD				
0 M	29.03 ± 15.53	56.39 ± 20.26	47.41 ± 19.14	/
6 M	15.05 ± 9.53	26.69 ± 20.72	22.04 ± 8.87	/
Improvement (%)	38.73 ± 38.03	54.55 ± 27.25	45.65 ± 26.42	0.3740
POEM				
0 M	11.53 ± 7.39	20.88 ± 6.48	16.69 ± 5.98	/
6 M	4.53 ± 3.16	8.13 ± 4.94	9.06 ± 3.84	/
Improvement (%)	49.68 ± 39.46	60.57 ± 21.83	43.68 ± 19.90	0.1827
Itch-NRS				
0 M	5.30 ± 2.37	7.75 ± 1.34	6.44 ± 2.76	/
6 M	2.68 ± 1.74	3.78 ± 2.01	2.50 ± 1.10	/
Improvement (%)	42.17 ± 52.64	51.91 ± 23.60	50.51 ± 33.17	0.6171
ADCT				
0 M	9.97 ± 6.18	16.88 ± 5.58	13.38 ± 6.03	/
6 M	4.03 ± 2.33	7.19 ± 4.37	5.06 ± 2.93	/
Improvement (%)	46.05 ± 45.19	59.02 ± 23.66	57.99 ± 29.29	0.5911
DLQI				
0 M	6.29 ± 4.68	12.75 ± 8.61	10.06 ± 5.00	/
6 M	3.91 ± 2.92	5.93 ± 5.12	6.44 ± 4.68	/
Improvement (%)	18.00 ± 58.10	45.52 ± 44.28	27.84 ± 44.72	0.2231

The improvement rates of clinical symptom scores were compared by Kruskal-Wallis test. AIT, allergen immunotherapy; SCORAD, scoring atopic dermatitis index; POEM, patient-oriented eczema measure; Itch-NRS, itch numerical rating scale; ADCT, atopic dermatitis control test; DLQI, dermatology life quality index. Improvement (%) = (scores at baseline - scores at 6 month)/scores at baseline∗100%

#### Safety

In the AIT group, a total of 10 patients (25.64%) experienced adverse reactions, the most common of which was local reaction, and 1 patient (2.56%) had chest tightness. Two patients (10.53%) in the dupilumab group developed eye symptoms (eye dryness, lacrimation or itching). In the AIT combined dupilumab group, 1 patient (5.26%) had conjunctival congestion, 2 patients (10.53%) had local reactions after AIT. The above adverse reactions were alleviated spontaneously or after symptomatic treatment (Table 3). No patients in the 3 groups experienced anaphylaxis.^24^

**Table 3:** Adverse reactions in 3 treatment groups.

Adverse reaction, n (%)	AIT group (n = 39)	Dupilumab group (n = 19)	AIT combined Dupilumab group (n = 19)
Local reaction	8 (20.51)	0	2 (10.53)
Aggravated skin lesions or pruritus	4 (10.26)	0	5 (26.32)
Eye symptoms	0	2 (10.53)	1 (5.26)
Chest tightness	1 (2.56)	0	0

Data are presented as numbers. AIT, allergen immunotherapy

#### Changes of tIgE and sIgE to HDM

**Fig. 3:**
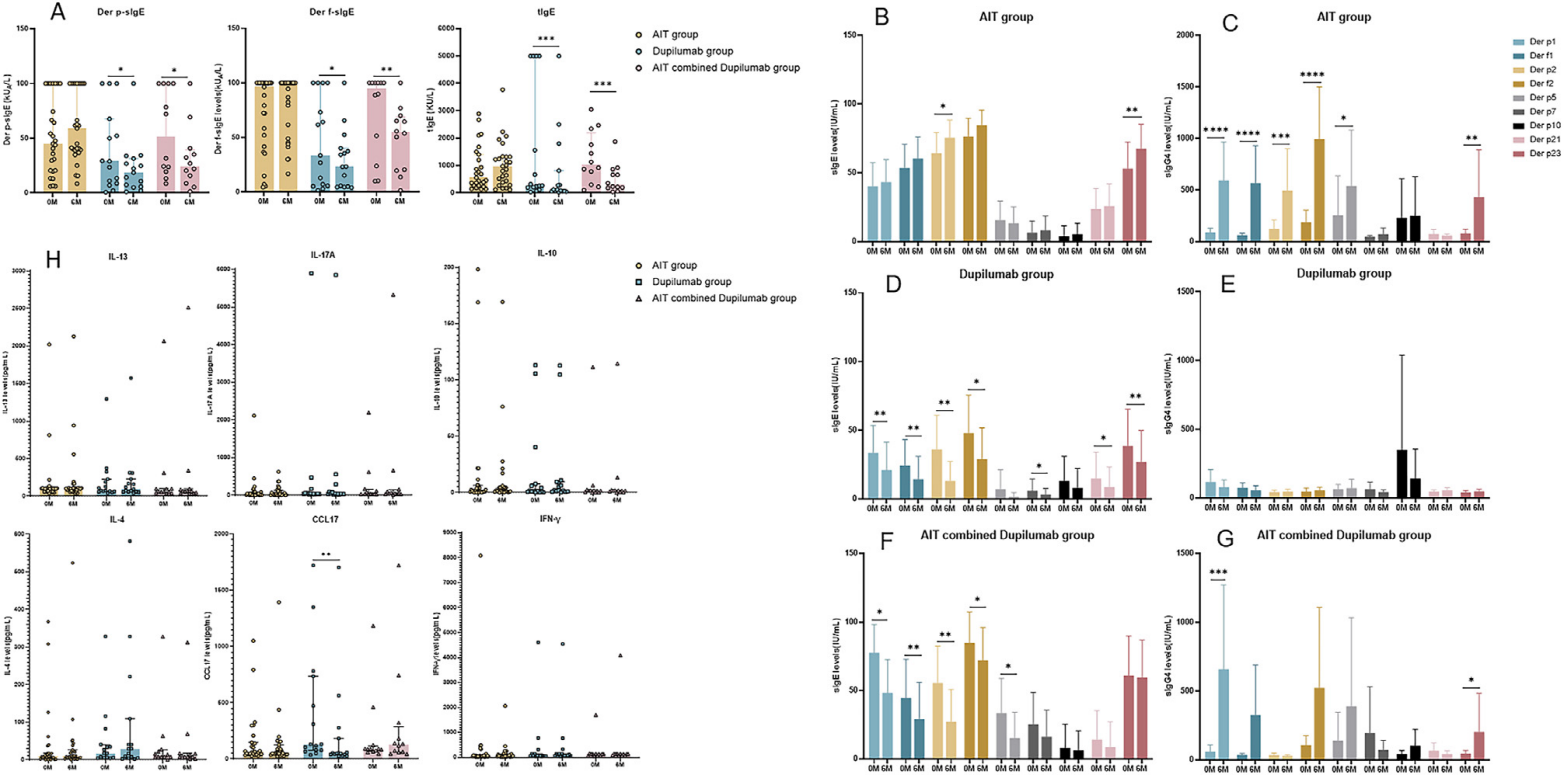
**Changes of serum immunological indicators in** 3 **treatment groups.** (A) The tIgE and sIgE to HDM at baseline and 6 months in 3 treatment groups. Levels of sIgE and sIgG_4_ to HDM components at baseline and 6 months in the (B, C) AIT group, (D, E) dupilumab group, and (F, G) AIT combined dupilumab group. (H) Levels of serum cytokines at baseline and 6 months in 3 treatment groups. Columns with error bars represent the mean with 95%CI for Fig. 3B–G and median with interquartile range for Fig. 3A and H, respectively. Changes of all the immunological indicators were analyzed by Wilcoxon test ∗P < 0.05; ∗∗P < 0.01; ∗∗∗P < 0.001; ∗∗∗∗P < 0.0001. AIT, allergen immunotherapy; *Der p*, *Dermatophagoides pteronyssinus*; *Der f*, *Dermatophagoides farina*; IL-4, Interleukin 4; IL-13, Interleukin 13; IL-17A, Interleukin 17A; IL-10, Interleukin 10; IFN-γ, Interferon-γ; CCL17, C–C Motif Chemokine Ligand 17; tIgE, total IgE; sIgE, specific IgE; sIgG_4_, specific IgG_4_

The levels of tIgE, *Der p*-sIgE and *Der f*-sIgE in the AIT group did not change, while the levels of tIgE, *Der p*-sIgE and *Der f*-sIgE in the dupilumab group and AIT combined dupilumab group significantly decreased after treatment (P < 0.05) (Fig. 3A).

#### Changes of sIgE and sIgG_4_ to HDM components

Serum *Der p2*-sIgE and *Der p23*-sIgE levels in the AIT group significantly increased after treatment (Fig. 3B). The changes of sIgE to HDM components in the dupilumab group and AIT combined dupilumab group were similar. *Der p1*-sIgE, *Der f1*-sIgE, *Der p2*-sIgE and *Der f2*-sIgE levels all decreased significantly after treatment (Fig. 3D, F). In the AIT group, the levels of *Der p1*-sIgG_4_, *Der f1*-sIgG_4_, *Der p2*-sIgG_4_, *Der f2*-sIgG_4_, *Der p5*-sIgG_4_, and *Der p23*-sIgG_4_ significantly increased after treatment (Fig. 3C). In the AIT combined dupilumab group, *Der p1*-sIgG_4_ and *Der p23*-sIgG_4_ levels significantly increased (Fig. 3G). There was no significant change in sIgG_4_ to HDM components in the dupilumab group (Fig. 3E). At baseline, there was no correlation observed between SCORAD scores and sIgE/sIgG_4_ levels (Table S1). Additionally, the change in SCORAD scores did not show any correlation with the change in sIgE/sIgG_4_ levels specific to HDM components across all groups (Table S2).

#### Changes of serum cytokines

Levels of serum cytokines in the AIT group and AIT combined dupilumab group did not change after treatment, while serum CCL17 levels in the dupilumab group decreased significantly from baseline (131.80 *vs* 51.79, P = 0.0026) (Fig. 3H).

#### Changes of CD4^+^T cell subsets

**Fig. 4:**
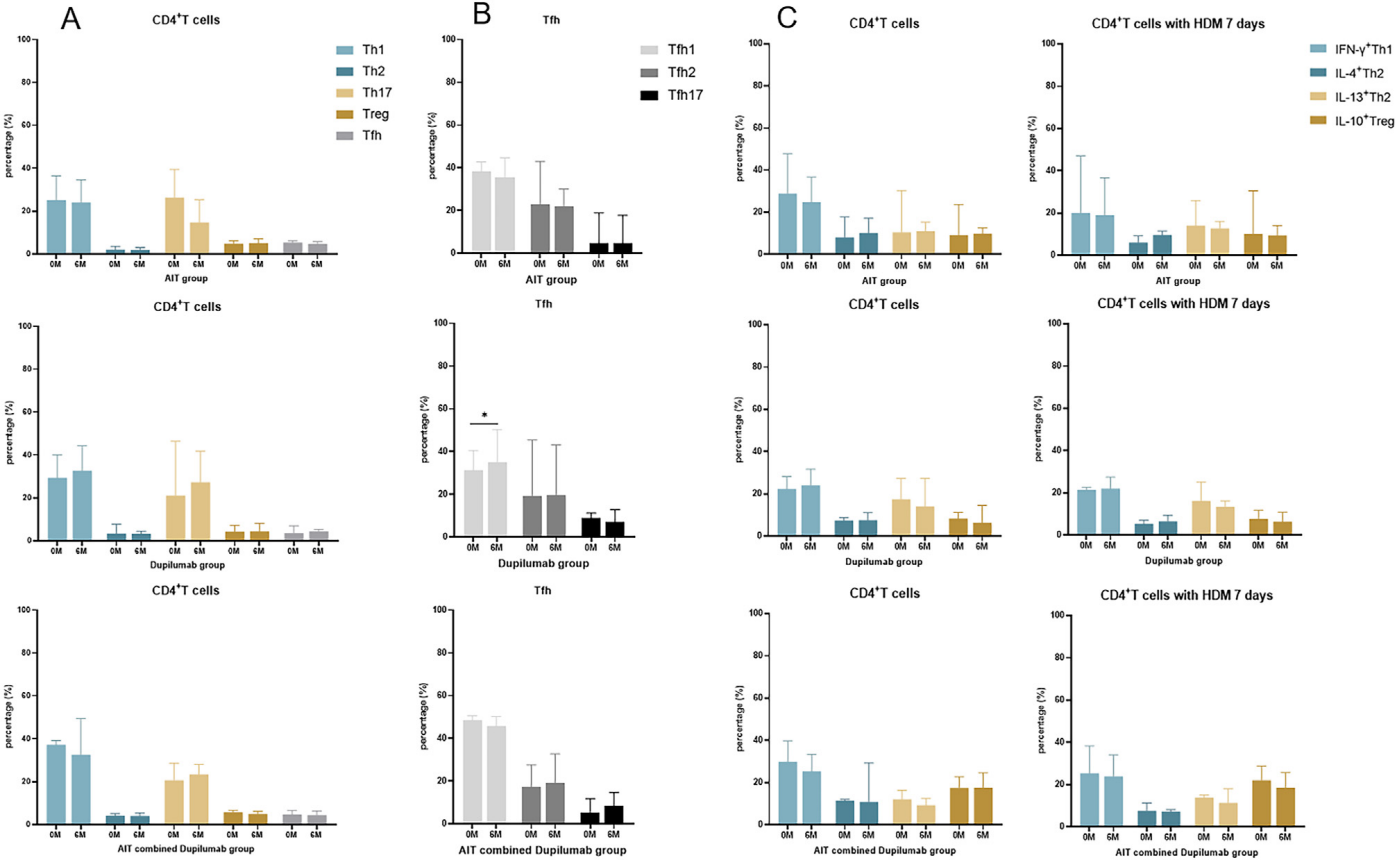
**Changes of CD4**^**+**^**T cells in** 3 **treatment groups.** The percentage of CD4^+^T cells at baseline and 6 months in the (A) AIT group, (B) dupilumab group, and (C) AIT combined dupilumab group. Wilcoxon test ∗P < 0.05. AIT, allergen immunotherapy; HDM, house dust mite; IL-4, Interleukin 4; IL-13, Interleukin 13; IL-10, Interleukin 10; IFN-γ, Interferon-γ

The proportion of Tfh1 cells (31.10% *vs* 34.80%, P = 0.0391) and ratios of Th1/Th2 and Th17/Th2 (8.19% *vs* 12.49%, P = 0.0156; 6.04% *vs* 8.96%, P = 0.0078) increased in the dupilumab group after treatment (Fig. 4B, Table S3B). There was no significant change in the proportion of CD4^+^T cells in the AIT group and AIT combined dupilumab group (Fig. 4A and B).

We further explored the changes of different cytokines expression in CD4^+^T cell subsets. The results showed that the proportion of IFN-γ^+^Th1 cells, IL-4^+^Th2 cells, IL-13^+^Th2 cells, and IL-10^+^Treg cells did not change after treatment with and without HDM stimulation in the 3 treatment groups (Fig. 4C).

**Fig. 5:**
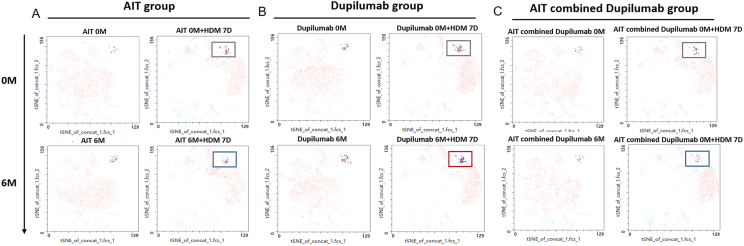
**The cluster of C2 (IL-4**^**+**^**IL-13**^**+**^**CRTH2**^**+**^**) with and without HDM stimulation at baseline and 6 months in the (A) AIT group, (B)**d**upilumab group, and (C) AIT combined**d**upilumab group.** The tSNE analysis visualized PBMCs incubated with HDM extracts for 7 days. AIT, allergen immunotherapy; HDM, house dust mite

**Fig. S2:**
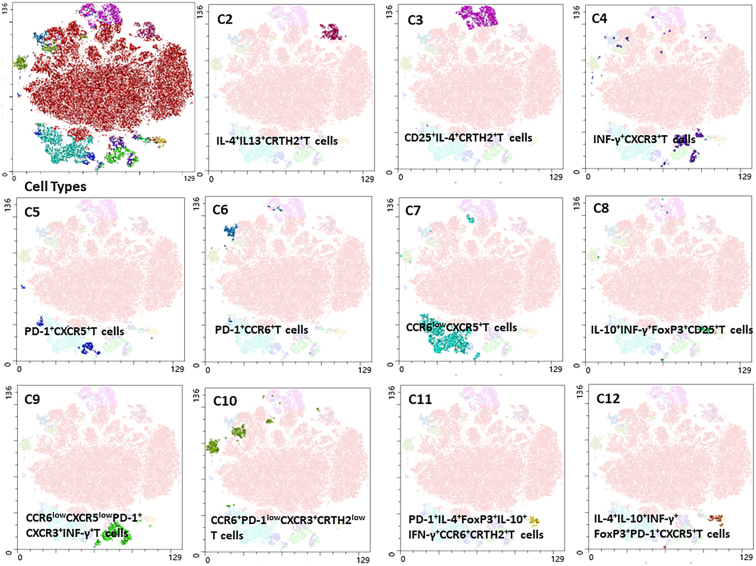
Cluster and visualize of CD3^+^CD4^+^live T cells among AD patients by tSNE analysis.

**Fig. S3:**
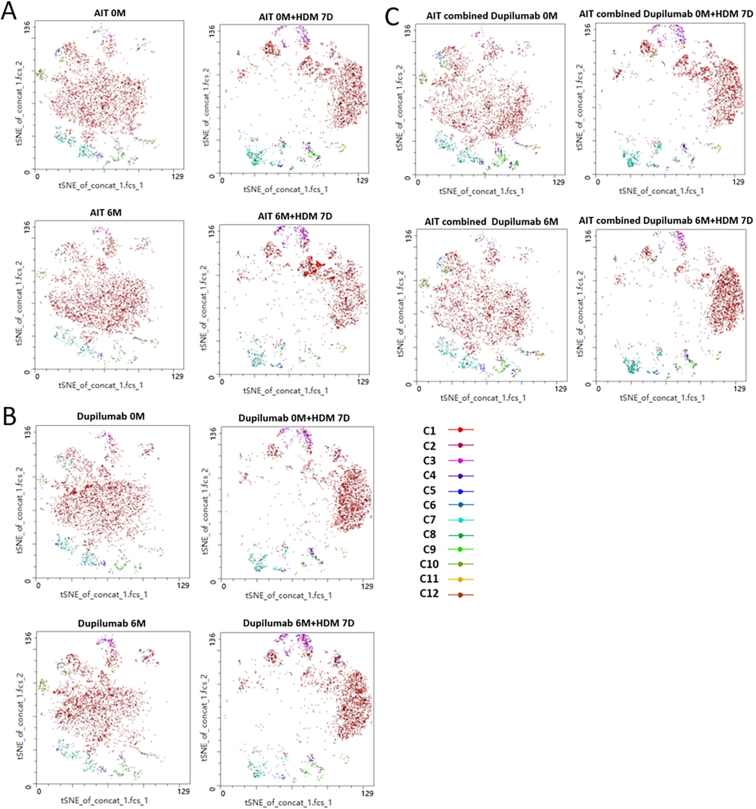
**The 12 clusters of CD3**^**+**^**CD4**^**+**^**live T cells with and without HDM stimulation at baseline and 6 months in the (A)AIT group, (B)Dupilumab group, and (C)AIT combined Dupilumab group.** AIT, allergen immunotherapy; HDM, house dust mite.

According to the expression profiles of CCR6, CXCR5, CXCR3, CRTH2, CD25, FoxP3, PD-1, IL-4, IL-13, IL-10, and IFN-γ, CD4^+^T cells were categorized into 12 clusters (Fig. S2). PBMCs collected at baseline were stimulated with HDM extracts (Fig. S3). Post-stimulation, there was an increase in the frequency of clusters of C2 (IL-4^+^IL-13^+^CRTH2^+^) and C3 (CD25^+^IL-4^+^CRTH2^+^), while cluster C8 (IL-10^+^IFN-γ^+^FoxP3^+^CD25^+^) and C10 (CCR6^+^PD-1^low^CXCR3^+^CRTH2^low^) decreased (Fig. S3). Following 6 months of treatment, an increase in the frequency of cluster C8 (IL-10^+^IFN-γ^+^FoxP3^+^CD25^+^) was observed across all groups (Fig. S3, Table S4). The frequency of cluster C9 (CCR6^low^CXCR5^low^PD-1^+^CXCR3^+^INF-γ^+^) and C10 (CCR6^+^PD-1^low^CXCR3^+^CRTH2^low^) decreased in the AIT group (Fig. S3A, Table S4), whereas cluster C10 increased in the dupilumab group (Fig. S3B, Table S4). Further analysis compared changes in PBMCs post-HDM stimulation among the 3 groups at 6 months. Notably, the frequency of cluster C2 (IL-4^+^IL-13^+^CRTH2^+^) remained unchanged in the AIT group (Fig. 5A) and the AIT combined dupilumab group (Fig. 5C), but increased in the dupilumab group (Fig. 5B).

#### Characteristics of good responders and poor responders in the 3 groups

**Fig. S4:**
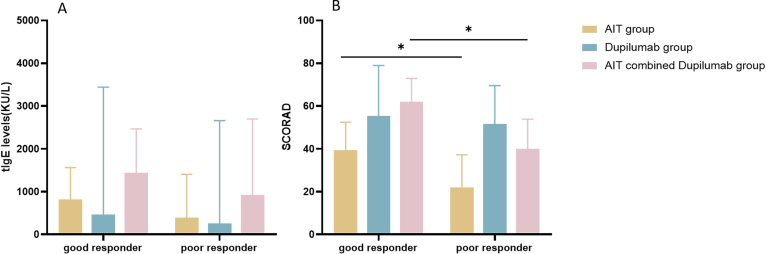
**The baseline (A) tIgE levels and (B) SCORAD scores of good responders(ΔSCORAD≥50%) and poor responders(ΔSCORAD<50%) in the AIT group, Dupilumab group, and AIT combined Dupilumab group.** Columns with error bars represented (A) the median with interquartile range and (B) mean with SD, respectively. Data were analyzed by Wilcoxon test ∗P < 0.05. AIT, allergen immunotherapy; SCORAD, scoring atopic dermatitis index; tIgE, total IgE.

Taking SCORAD score improvement of 50% (SCORAD-50) after 6 months of treatment as the standard.^25^^,^^26^ AD patients in the 3 treatment groups were divided into good responders (ΔSCORAD≥50%) and poor responders (ΔSCORAD<50%). There was no significant difference in baseline tIgE level between good responders and poor responders in the 3 groups (Fig. S4A). Baseline SCORAD scores of good responders in the AIT group and AIT combined dupilumab group were significantly higher than poor responders (39.36 ± 13.10 *vs* 22.02 ± 15.17, P = 0.0037; 62.06 ± 10.87 *vs* 39.98 ± 13.87, P = 0.0303) (Fig. S4B). The levels of CCL17 and IL-10 of good responders were significantly higher than poor responders in the AIT group and dupilumab group (Table S5A, Table S5B).

## Discussion

In our study, significant symptom improvement was observed in patients across the AIT group, the dupilumab group, and the combination therapy group post-treatment, indicating the efficacy of both AIT and dupilumab in treating AD. AIT led to a notable increase in IgG_4_ levels, while dupilumab treatment resulted in an elevation of the Th1/Th2 ratio and a significant decrease in IgE levels from an immunological standpoint. PBMCs from the AIT group displayed suppressed proliferation of IL-4^+^CRTH2^+^T cells after stimulation with HDM extracts, a response not seen with dupilumab. Our study reaffirms the effectiveness of AIT and dupilumab in treating AD, each with distinct mechanisms of action. However, there was a lack of apparent synergistic clinical benefits in the combination therapy approach, which also did not mitigate the safety risks associated with AIT.

Our results further confirm the efficacy and safety of dupilumab in AD. Dupilumab has shown significant improvement in clinical symptoms and quality of life in moderate to severe AD patients, both in randomized controlled trials and real-world studies.^27^^,^^28^ Our research also indicates the favorable effects of dupilumab in treating AD, with a proportion exceeding 60% of patients experiencing a 50% reduction in SCORAD scores, and a very low incidence of adverse reactions, with only 10% of patients experiencing conjunctivitis, and no other adverse reactions observed. Our study reiterates that dupilumab is a potent weapon in the treatment of AD. Further analysis reveals that AD patients with higher baseline blood levels of CCL17 and IL-10 exhibit a higher response rate to dupilumab, suggesting these 2 molecules could serve as potential biomarkers for assessing dupilumab treatment efficacy in clinical practice. Previous studies also reported that the decrease of CCL17 after dupilumab treatment was associated with the improvement of EASI and itch socre.^29^^,^^30^ However, larger clinical studies are needed to validate our findings.

Compared to the high consistency of clinical efficacy results of dupilumab in different regions and populations, the role of AIT in AD remains controversial. Previous studies have shown that AIT, including subcutaneous and sublingual routes, can improve SCORAD scores in AD patients, but with minimal changes in quality of life scores and itch scores.^31^, ^32^, ^33^, ^34^ One previous study have found that AIT did not improve the clinical symptoms of AD patients.^35^ However, a recent meta-analysis suggests the benefits of AIT for AD patients.^26^ Due to the heterogeneity of research results and the lack of high-quality RCT studies, some guidelines currently do not recommend AIT as a treatment strategy for AD, although they also note that AIT is not contraindicated for AD.^36^^,^^37^ Our study found a significant decrease in SCORAD scores in AD patients after receiving AIT, confirming that HDM-sensitized AD patients can benefit from AIT, with higher response rates to AIT observed in patients with higher baseline SCORAD scores, which was consistent with previous studies.^25^ In terms of adverse reaction rates, the main adverse reactions of AIT in our study were local and no systemic reaction was observed, indicating the safety and efficacy of AIT for AD patients.

Considering the favorable and rapid symptom control effect of dupilumab and the slower onset but disease-modifying and long-term efficacy of AIT, we also explored whether a combination of dupilumab and AIT could have a synergistic therapeutic effect in AD. We observed a significant decrease in SCORAD scores from baseline in the combination therapy group, with mild treatment adverse reactions, indicating that dupilumab combined with AIT is also safe and effective in treating AD.

While there were no differences in age, gender, and duration of illness, the baseline severity of AD varied among the AIT group, dupilumab group, and combination group due to non-randomized assignment. To assess treatment effects across the 3 groups, we compared the reduction proportions of symptom scores. We found that the magnitude of improvement in AD patients in these 3 groups was similar, particularly with the combination group not demonstrating a greater advantage over AIT or dupilumab, consistent with previous research findings.^38^ As both the dupilumab group and the combination group exhibited similar severity of symptoms at baseline, ensuring comparability, we speculate that the lack of greater advantage in the combination group over the dupilumab group may be attributed to transient symptom exacerbation in the early stage of AIT as indicated in previous studies,^39^ offsetting potential synergistic effects between dupilumab and AIT. However, a small sample size study showed significant improvement in symptoms of AD patients with poor response to AIT or dupilumab after receiving combination therapy.^40^ Additionally, several studies indicate that combination therapy can improve symptoms and reduce the incidence of adverse reactions in allergic rhinitis patients.^38^^,^^41^ Therefore, we also anticipate large-scale, long-term RCT studies to confirm whether AIT and dupilumab can synergistically act in the treatment of AD.

To further investigate the immunological mechanisms of AIT and dupilumab in AD, we compared the changes in serum cytokines, HDM-specific IgE, IgG_4_ antibodies, and T cell subsets before and after treatment in 3 groups. We found that the total IgE and HDM-specific IgE levels in the AIT group showed no difference before and after treatment, while the HDM-specific IgG_4_ levels significantly increased. In contrast, the dupilumab group showed a significant decrease in total IgE and HDM-specific IgE levels after treatment, with no significant change in IgG_4_ levels. These observations suggested different mechanisms of action for AIT and dupilumab.^32^^,^^42^ However, a previous study has demonstrated an increase in HDM-sIgG_4_ levels after 3 years of dupilumab treatment.^43^ We hypothesize that the impact of dupilumab on the immune system, such as IgG antibodies production, may be linked to the duration of treatment. Yet, a recent study^44^ found that in AD patients treated with dupilumab, there was no significant change in the production of IgG and IgG_4_ during a 144-week observation period, which is consistent with our findings. In the combination therapy group, IgE levels decreased while IgG_4_ levels increased, exhibiting characteristics of both AIT and dupilumab. These findings indicated that immunomodulatory effects of AIT, such as the regulatory T cell and IL-10 pathway, play a more important role in IgG_4_ production, which appears unaffected by dupilumab.

Since antibody production originates from B cells and the class switching of antibodies by B cells depends on cytokines, particularly type 2 cytokines like IL-4, IL-13, and IL-21,^45^ we further compared the changes in cytokines among the 3 groups before and after treatment. We observed no significant differences in IL-4, IL-13, and other cytokines in the 3 groups before and after treatment. We only found a decrease in CCL17, a cytokine related to the severity of AD, in the dupilumab treatment group, which aligns with the improvement in symptoms.^29^^,^^46^ We speculate that the levels of serum type 2 inflammatory cytokines may not necessarily reflect the local skin inflammatory response, leading to the lack of statistically significant changes observed before and after treatment in the 3 groups.

Given the significant role of T cells, especially Tfh cells, in assisting B cells to produce IgE antibodies,^47^ we also compared the changes in peripheral blood T cell subsets. We observed that there were no significant changes in Th1, Th2, Treg, and Tfh cell subsets after 6 months of AIT. These results were in line with previous research conducted by Hajdu et al.^33^ They also found no significant change in Th1 and Th2 cells in peripheral blood after AIT. But a decrease in skin infiltrating DCs and CD4^+^T cells was observed after treatment suggesting that the inflammation reflected by local tissue changes was more significant than that in blood. In the dupilumab group, the proportions of T cell subsets showed no significant changes before and after treatment, but the Th1/Th2 ratio significantly increased post-treatment, possibly due to dupilumab's inhibition of the Th2 subset. The changes in T cell subsets in the combination therapy group were similar to those in the AIT group, and no synergistic effect between dupilumab and AIT was observed.

Using tSNE technology to analyze T lymphocyte changes, we can thoroughly identify alterations in cell subpopulations throughout the course of AD treatment. We found that the frequency of cluster C2 (IL-4^+^IL-13^+^CRTH2^+^) increased after HDM stimulation at baseline. However, after treatment and subsequent HDM stimulation, only the dupilumab group showed an increase of cluster C2, while there was no change in the AIT group and the combination therapy group. This suggests that AIT can inhibit the proliferation response of Th2 cells following HDM stimulation, while dupilumab lacks this effect, further confirming the disease-modifying function of AIT in inducing long-term immune tolerance to allergens in individuals with AD. Moreover, the frequency of cluster C8 (IL-10^+^IFN-γ^+^FoxP3^+^CD25^+^), representing a Treg cell subgroup expressing IL-10 and IFN-γ, increased in all groups post-treatment. While AIT is known to induce Tregs, the impact of dupilumab on Tregs remains unclear. Our findings suggest that dupilumab holds promise in sustaining remission in AD, with this regulatory effect potentially linked to the treatment duration. These results align with Bakker's findings, where the proportion of CD4^+^CD25^+^FoxP3^+^T cells in AD patients increased after 52 weeks of dupilumab treatment, showing no significant difference compared to baseline at 16 weeks.^48^ However, the key pathways of AIT and dupilumab on AD still require further investigation.

There are several limitations in our study. Firstly, the duration of AIT treatment in the study was relatively short, whereas optimal efficacy typically requires at least 3 years. The long-term effectiveness and immunological mechanisms of both AIT and dupilumab were not investigated in this research. Secondly, the absence of an AD control group solely on topical medications hinders the accurate interpretation of the immunological results as effects of AIT/dupilumab treatment. Nonetheless, previous RCT studies have indicated that natural exposure and topical treatments do not impact serum cytokines and immunoglobulins in AD patients,^30^^,^^49^ suggesting minimal influence on our study's conclusions. Thirdly, the study did not verify the causal relationship between HDM and AD, despite the patients exhibiting relatively high IgE levels and half of them having respiratory comorbidities. HDM provocation tests or patch tests were not conducted. Additionally, the treatments given to AD patients were not randomly assigned, leading to an imbalance in the severity of AD, particularly evident in the baseline SCORAD scores. Consequently, direct comparisons of score changes were challenging, with comparisons made based on percentage changes, potentially introducing bias. Lastly, the study's findings are limited by a small sample size, with a few patients dropping out before completion. Future research should involve large randomized controlled trials to effectively address these limitations.

In conclusion, AIT, dupilumab, and the combination treatment improved clinical symptoms and quality of life in AD patients. AIT promoted allergen-specific IgG_4_ production, while dupilumab reduced allergen-specific IgE synthesis. The combination of AIT and dupilumab exhibited the immunological parameter changes characteristic of both treatments but did not result in a significantly greater improvement in AD symptoms. AIT can inhibit the proliferation response of Th2 cells following HDM stimulation. Dupilumab lacks this disease-modifying effect but presents a moderating effect on T cell responses with an increase in Th1/Th2 ratio.

## Abbreviations

AD, atopic dermatitis; AIT, allergen immunotherapy; AR, allergic rhinitis; *Der p*, *Dermatophagoides pteronyssinus*; *Der f*, *Dermatophagoides farina*; DLQI, dermatology life quality index; EASI, eczema area and severity index; HDM, house dust mite; SCIT, subcutaneous immunotherapy; SLIT, sublingual immunotherapy; t-SNE, T-distributed stochastic neighbor embedding; SCORAD, scoring atopic dermatitis index; POEM, patient-oriented eczema measure; Itch-NRS, itch numerical rating scale.

## Availability of data and materials

The datasets used and/or analyzed during the current study are available from the corresponding author on reasonable request.

## Author contributions

YL and ZR conceived and designed the study. YL and ZR supervised the study. LJ and YL did the statistical analysis. LJ collected the information about the participants. XQ, JQ, HN, LW, YY, MD, LL, FY, CH performed the clinical tests and collected the data. YL and LJ completed the serological and cytological experiment. LJ drafted the manuscript.

## Ethics statement

The study protocol was approved by the Independent Ethical Committee of Tongji Hospital (TJ IRB20231112). Each participant received detailed information about the study and study methods and provided written informed consent for their participation.

## Consent for publication

All authors contributed to acquisition, analysis, or interpretation of data, revised the report, and approved the final version before submission.

## Funding

This work was supported by the Fundamental Research Project of Wuhan Science and Technology Bureau (No. 2023020201010157).

## Declaration of competing interest

The authors declare that they have no competing interests.
