# Supplementary material for: Allergen immunotherapy and dupilumab in atopic dermatitis: Clinical efficacy and disparities in immunological indicators
Source: World Allergy Organ J. 2025 Mar 12;18(3):101043. doi: 10.1016/j.waojou.2025.101043 (PMC11946805; doi:10.1016/j.waojou.2025.101043)
Supplement: Multimedia component 1 [file mmc1.docx]

**Table S1. Correlation analysis of baseline sIgE/sIgG_4_ to HDM components and SCORAD scores.**

| sIgE/sIgG_4_ | R^2^ | 95%CI | *P* value |
| --- | --- | --- | --- |
| *Der p1* | 0.1508 | -0.1299 to 0.4091 | 0.2764 |
| *Der f1* | -0.0600 | -0.3349 to 0.2243 | 0.6728 |
| *Der p2* | -0.1230 | -0.3852 to 0.1576 | 0.3757 |
| *Der f2* | 0.0465 | -0.2317 to 0.3177 | 0.7384 |
| *Der p5* | 0.0856 | -0.1942 to 0.3526 | 0.5382 |
| *Der p7* | 0.2840 | 0.0094 to 0.5187 | 0.0574 |
| *Der p*1*0* | 0.0985 | -0.1872 to 0.3689 | 0.4872 |
| *Der p21* | 0.0116 | -0.2672 to 0.2885 | 0.9345 |
| *Der p23* | -0.0973 | -0.3629 to 0.1829 | 0.4839 |

The correlations between baseline SCORAD scores and levels of sIgE/sIgG_4_ to HDM components were analyzed by Spearman rank test. AIT, allergen immunotherapy; *Der p*, *Dermatophagoides pteronyssinus*; *Der f*, *Dermatophagoides farina.*

**Table S2. Correlation analysis of changes of sIgE/sIgG_4_(△sIgE/sIgG_4_) to HDM components and changes of SCORAD scores (△SCORAD).**

| △sIgE/sIgG_4_ | R^2^ | 95%CI | *P* value |
| --- | --- | --- | --- |
| **AIT group** | | | |
| *Der p1* | -0.1221 | -0.4889 to 0.2814 | 0.5440 |
| *Der f1* | -0.1608 | -0.5316 to 0.2618 | 0.4427 |
| *Der p2* | -0.1768 | -0.5366 to 0.2375 | 0.3877 |
| *Der f2* | -0.2613 | -0.5911 to 0.1434 | 0.1880 |
| *Der p5* | -0.3322 | -0.6394 to 0.0665 | 0.0905 |
| *Der p7* | -0.1699 | -0.5316 to 0.2442 | 0.4066 |
| *Der p*1*0* | 0.03923 | -0.3722 to 0.4378 | 0.8523 |
| *Der p21* | -0.1731 | -0.5406 to 0.2500 | 0.4080 |
| *Der p23* | -0.3797 | -0.6705 to 0.0121 | 0.0507 |
| **Dupilumab group** | | | |
| *Der p1* | 0.1464 | -0.4095 to 0.6231 | 0.6024 |
| *Der f1* | -0.2429 | -0.6807 to 0.3227 | 0.3820 |
| *Der p2* | -0.3821 | -0.7553 to 0.1780 | 0.1607 |
| *Der f2* | -0.4464 | -0.7867 to 0.1019 | 0.0972 |
| *Der p5* | -0.2176 | -0.6802 to 0.3690 | 0.4542 |
| *Der p7* | -0.04286 | -0.5549 to 0.4927 | 0.8828 |
| *Der p*1*0* | 0.4560 | -0.1448 to 0.8112 | 0.1198 |
| *Der p21* | -0.4964 | -0.8100 to 0.0379 | 0.0623 |
| *Der p23* | -0.3643 | -0.7462 to 0.1980 | 0.1824 |
| **AIT combined Dupilumab group** | | | |
| *Der p1* | 0.5245 | -0.0899 to 0.8497 | 0.0839 |
| *Der f1* | -0.2273 | -0.7374 to 0.4479 | 0.5034 |
| *Der p2* | -0.1538 | -0.6792 to 0.4758 | 0.6353 |
| *Der f2* | -0.2238 | -0.7164 to 0.4178 | 0.4851 |
| *Der p5* | -0.2517 | -0.7306 to 0.3930 | 0.4303 |
| *Der p7* | -0.0839 | -0.6392 to 0.5288 | 0.8004 |
| *Der p*1*0* | 0.3007 | -0.3473 to 0.7543 | 0.3424 |
| *Der p21* | -0.2937 | -0.7510 to 0.3540 | 0.3545 |
| *Der p23* | 0.1329 | -0.4922 to 0.6675 | 0.6832 |

The correlations between changes of SCORAD scores and changes of sIgE/sIgG_4_ to HDM components levels were analyzed by Spearman rank test.

△sIgE/sIgG_4_= (sIgE/sIgG_4_ 6M- sIgE/sIgG_4_ 0M)/ sIgE/sIgG_4_ 0M; △SCORAD=(SCORAD 6M-SCORAD 0M)/SCORAD 0M. AIT, allergen immunotherapy; *Der p*, *Dermatophagoides pteronyssinus*; *Der f*, *Dermatophagoides farina.*

**Table S3A. The ratio of CD4^+^T cell subsets at baseline and 6 months in AIT group**

|  | 0M | 6M | P value |
| --- | --- | --- | --- |
| Th1/Th2 | 12.76 (8.05, 16.00) | 10.38 (8.24, 14.03) | 0.3008 |
| Th1/Th17 | 1.14 (0.79, 1.40) | 1.35 (0.82, 2.21) | 0.5703 |
| Th17/Th2 | 9.81 (6.08, 14.09) | 7.92 (6.42, 9.25) | 0.1758 |
| Tfh1/Tfh2 | 1.71 (0.46, 3.07) | 2.08 (0.98, 2.71) | 0.4258 |
| Tfh1/Tfh17 | 5.35 (1.29, 10.82) | 7.02 (1.53, 16.03) | 0.1484 |
| Tfh2/Tfh17 | 2.74 (1.34, 4.85) | 4.11 (1.09, 8.79) | 0.5781 |

**Table S3B. The ratio of CD4^+^T cell subsets at baseline and 6 months in Dupilumab group**

|  | 0M | 6M | P value |
| --- | --- | --- | --- |
| Th1/Th2* | 8.19 (3.13, 12.54) | 12.49 (5.87, 15.78) | **0.0156** |
| Th1/Th17 | 1.02 (0.79, 1.36) | 1.18 (0.71, 1.47) | 0.6406 |
| Th17/Th2* | 6.04 (2.79, 12.22) | 8.96 (6.22, 17.93) | **0.0078** |
| Tfh1/Tfh2 | 2.13 (0.28, 2.50) | 1.64 (0.58, 2.77) | 0.5469 |
| Tfh1/Tfh17 | 3.91 (1.10, 5.20) | 5.11 (1.92, 11.24) | 0.0781 |
| Tfh2/Tfh17 | 2.09 (1.87, 3.57) | 3.22 (2.67, 4.06) | 0.2500 |

**Table S3C. The ratio of CD4^+^T cell subsets at baseline and 6 months in AIT combined Dupilumab group**

|  | 0M | 6M | P value |
| --- | --- | --- | --- |
| Th1/Th2 | 9.35 (5.94, 14.97) | 8.72 (8.21, 12.13) | >0.9999 |
| Th1/Th17 | 1.25 (0.97, 1.98) | 1.55 (1.14, 1.88) | 0.7500 |
| Th17/Th2 | 5.75 (4.04, 14.38) | 6.42 (4.43, 9.99) | 0.8125 |
| Tfh1/Tfh2 | 2.90 (1.36, 4.51) | 2.46 (1.07, 3.87) | 0.0625 |
| Tfh1/Tfh17 | 8.90 (5.21, 10.21) | 5.61 (2.58, 9.91) | 0.1250 |
| Tfh2/Tfh17 | 2.76 (1.55, 4.90) | 2.28 (1.43, 4.42) | 0.4375 |

Data are presented as median and interquartile range (IQR). Wilcoxon test *p < 0.05. AIT, allergen immunotherapy; Th1, type 1 helper T; Th2, type 2 helper T; Th17, type 17 helper T; Tfh, follicular helper T; Tfh1, type 1 Tfh; Tfh2, type 2 Tfh; Tfh17, type 17 Tfh.

**Table S4. The frequency of 12 clusters of CD4^+^T cell at baseline and 6 months in three treatment groups.**

| **clusters** | **phenotype** | **AIT group** | | | | **AIT combined Dupilumab group** | | | | **Dupilumab group** | | | |
| --- | --- | --- | --- | --- | --- | --- | --- | --- | --- | --- | --- | --- | --- |
|  |  | **0M** | | **6M** | | **0M** | | **6M** | | **0M** | | **6M** | |
|  |  | HDM(-) | HDM(+) | HDM(-) | HDM(+) | HDM(-) | HDM(+) | HDM(-) | HDM(+) | HDM(-) | HDM(+) | HDM(-) | HDM(+) |
| **C1** | naïve T cell | 5.67% | 5.1% | 6.1% | 5.47% | 5.38% | 5.18% | 5.54% | 5.34% | 5.83% | 5.46% | 5.59% | 5.19% |
| **C2** | CRTH2^+^IL-4^+^IL13^+^T cell | 1.81% | 7.23% | 4.34% | 5.18% | 3.01% | 6.39% | 3.01% | 4.22% | 6.39% | 11.33% | 8.92% | 14.46% |
| **C3** | CD25^+^IL-4^+^CRTH2^+^T cell | 1.57% | 7.87% | 2.35% | 13.1% | 1.32% | 10.58% | 1.69% | 8.4% | 2.47% | 10.96% | 5.11% | 17.92% |
| **C4** | CXCR3^+^INF-γ^+^T cell | 4.32% | 7.05% | 2.73% | 2.59% | 9.5% | 7.91% | 5.76% | 5.76% | 3.88% | 6.62% | 5.04% | 4.46% |
| **C5** | CXCR5^+^PD-1^+^T cell | 6.19% | 3.75% | 5.25% | 3.75% | 8.82% | 2.63% | 9.01% | 4.5% | 6.75% | 3.19% | 6.19% | 3.56% |
| **C6** | CCR6^+^PD-1^+^T cell | 8.61% | 5% | 3.61% | 2.78% | 11.11% | 3.33% | 7.22% | 3.61% | 7.78% | 2.22% | 5.28% | 3.33% |
| **C7** | CCR6^low^CXCR5^+^T cell | 5.78% | 8.72% | 3.44% | 5.97% | 5.94% | 8.31% | 5.44% | 7.97% | 4.9% | 5.22% | 3.78% | 4.1% |
| **C8** | FoxP3^+^CD25^+^IL-10+INF-γ^+^T cell | 5.56% | 2.78% | 8.8% | 1.39% | 9.26% | 3.24% | 9.72% | 3.24% | 2.31% | 1.39% | 4.63% | 0.00% |
| **C9** | CCR6^low^CXCR5^+^PD-1^+^CXCR3^+^INF-γ^+^T cell | 6.71% | 8.85% | 2.89% | 2.78% | 9.66% | 3.82% | 7.06% | 7.00% | 4.57% | 4.45% | 5.84% | 4.68% |
| **C10** | CCR6^+^PD-1^+^CXCR3^+^CRTH2^+^T cell | 9.35% | 1.8% | 3.34% | 0.86% | 9.01% | 2.32% | 9.52% | 0.77% | 4.97% | 0.43% | 11.06% | 0.51% |
| **C11** | PD-1^+^IL-4^+^FoxP3^+^IL-10^+^ IFN-γ^+^CCR6^+^CRTH2^+^T cell | 2.33% | 12.29% | 2.66% | 7.97% | 5.98% | 15.28% | 9.63% | 5.98% | 2.33% | 3.65% | 1.00% | 3.99% |
| **C12** | IL-4^+^IL-10^+^INF-γ^+^ FoxP3^+^PD-1^+^CXCR5^+^T cells | 5.83% | 5.52% | 5.83% | 3.37% | 7.36% | 3.99% | 9.2% | 3.07% | 3.37% | 4.6% | 3.07% | 0.92% |

Clusters of CD3^+^CD4^+^live T cells among AD patients in three treatment groups at baseline and 6 months by tSNE analysis. HDM(+), PBMCs stimulated with HDM; HDM(-), PBMCs without HDM stimulation. AIT, allergen immunotherapy.

**Table S5A. Baseline cytokine levels of good responders(∆SCORAD≥50%) and poor responders(∆SCORAD<50%) in AIT group.**

|  | good responders | poor responders | *P* value |
| --- | --- | --- | --- |
| IL-4, pg/mL | 12.50 (7.73, 104.40) | 7.55 (1.67, 17.14) | 0.1225 |
| IL-13, pg/mL | 106.30 (59.66, 199.20) | 100.80 (70.23, 110.20) | 0.4779 |
| IL-17A, pg/mL | 36.75 (11.10, 137.00) | 38.29 (4.68, 43.16) | 0.3728 |
| CCL17*, pg/mL | 136.20 (53.84, 320.80) | 49.77 (43.05, 70.16) | **0.0365** |
| IL-10*, pg/mL | 4.09 (1.64, 17.98) | 0.86 (0.34, 4.35) | **0.0186** |
| IFN-γ, pg/mL | 64.95 (38.69, 377.30) | 47.10 (24.89, 63.04) | 0.0926 |

**Table S5B. Baseline cytokine levels of good responders(∆SCORAD≥50%) and poor responders(∆SCORAD<50%) in Dupilumab group.**

|  | good responders | poor responders | *P* value |
| --- | --- | --- | --- |
| IL-4, pg/mL | 34.13 (14.88, 91.25) | 6.24 (5.10, 17.68) | 0.0753 |
| IL-13, pg/mL | 119.60 (68.60, 340.00) | 67.82 (52.16, 152.90) | 0.1645 |
| IL-17A, pg/mL | 54.22 (33.13, 243.80) | 42.46 (35.20, 44.56) | 0.4396 |
| CCL17*, pg/mL | 392.30 (119.20, 925.10) | 78.33 (59.12, 112.10) | **0.0280** |
| IL-10*, pg/mL | 5.17 (0.89, 56.79) | 0.92 (0.27, 1.55) | **0.0420** |
| IFN-γ, pg/mL | 62.88 (35.68, 427.70) | 59.11 (50.45, 103.70) | 0.8591 |

**Table S5C. Baseline cytokine levels of good responders(∆SCORAD≥50%) and poor responders(∆SCORAD<50%) in AIT combined Dupilumab group.**

|  | good responders | poor responders | *P* value |
| --- | --- | --- | --- |
| IL-4, pg/mL | 14.03 (8.00, 21.98) | 9.14 (7.55, 46.01) | >0.9999 |
| IL-13, pg/mL | 62.74 (47.88, 102.60) | 68.96 (48.60, 205.00) | >0.9999 |
| IL-17A, pg/mL | 35.54 (35.41, 172.40) | 44.20 (32.70, 354.30) | 0.9646 |
| CCL17, pg/mL | 76.06 (59.46, 93.30) | 89.56 (87.30, 293.30) | 0.3434 |
| IL-10, pg/mL | 1.79 (0.15, 2.59) | 1.57 (0.40, 4.17) | 0.9116 |
| IFN-γ, pg/mL | 65.43 (50.57, 138.40) | 51.92 (39.07, 117.50) | 0.5303 |

Data are presented as median and interquartile range (IQR). Wilcoxon test *p < 0.05. IL-4, Interleukin 4; IL-13, Interleukin 13; IL-17A, Interleukin 17A; IL-10, Interleukin 10; IFN-γ, Interferon-γ; CCL17, C-C Motif Chemokine Ligand 17.
